# Treatment and long-term follow-up of a cat with leishmaniosis

**DOI:** 10.1186/s13071-019-3388-9

**Published:** 2019-03-26

**Authors:** Emanuele Brianti, Nunziata Celi, Ettore Napoli, Jessica M. Abbate, Francesca Arfuso, Gabriella Gaglio, Roberta Iatta, Salvatore Giannetto, Marina Gramiccia, Domenico Otranto

**Affiliations:** 1 Department of Veterinary Sciences, University of Messina, Polo University Annunziata, Messina, Italy; 2 Veterinary Practitioner, Messina, Italy; 3 Department of Veterinary Medicine, University of Bari, St. prov. per Casamassima km 3, Bari, Italy; 4 Unit of Vector-Borne Diseases, Department of Infectious Diseases, Istituto Superiore di Sanità, Rome, Italy

**Keywords:** Feline leishmaniosis, *Leishmania infantum*, Cat, Treatment, Allopurinol, PCR-RFLP, ITS1, HSP70, Sequencing analysis

## Abstract

**Background:**

*Leishmania* infection in cats is being increasingly reported in endemic areas. Nevertheless, only a few clinical cases have been described in cats, and even fewer have provided information on the response to treatment and a proper follow-up. Here we report a case of feline leishmaniosis not associated with any other disease or co-infection and document its response to allopurinol treatment and long-term follow-up data.

**Results:**

A 6-year-old domestic shorthair female cat was referred for nodular blepharitis, mucocutaneous ulcerative lesions of the mouth and lymph node enlargement. The cat was moderately anaemic, hyperglobulinaemic and tested negative for feline leukaemia virus and feline immunodeficiency virus. Fine needle aspirates of nodules and mucocutaneous lesions showed the presence of numerous amastigote forms of *Leishmania*. *Leishmania* infection was further confirmed by serology (IFAT test, 1:640) and real-time PCR (RT-PCR) on blood and conjunctival swabs. The cat was treated with allopurinol (20 mg/kg SID), which was clinically effective, although the cat remained *Leishmania-*positive in serology and RT-PCR on blood and conjunctival swabs. Allopurinol treatment was interrupted after seven months because of the healing of all lesions and lack of compliance by the owner. After two years, the cat relapsed displaying almost the same clinical signs and clinicopathological alterations. On this occasion, the parasite was isolated by culture and identified as belonging to *L. infantum*. Allopurinol treatment was started again but was interrupted several times because of the itching side effect observed. The cat worsened progressively and died two months after the relapse without any chance to shift the treatment to another molecule (e.g. meglumineantimoniate or miltefosine).

**Conclusions:**

Out of all documented cases of feline leishmanosis, the present case has the longest follow-up period and it is one of the few in which the parasite was isolated and identified. It further confirms the potential progression of *Leishmania* infection to disease in cats even in the absence of comorbidities. Veterinarians practicing in endemic areas should be aware of this susceptibility, properly include feline leishmaniosis in the differential diagnosis and propose preventative measures to those cats at risk.

## Background

Leishmaniosis, caused by *Leishmania infantum*, is one of the most important vector-borne zoonotic diseases worldwide [1]. Dogs are regarded as the main reservoir hosts of *Leishmania infantum* in endemic areas but the role of other domestic and sylvatic animals in the epidemiology of the infection has recently gained prominence [2–4].

Infection by *L. infantum* in cats has been increasingly reported in the same areas where canine leishmaniosis is endemic [4, 5]. Although the proportion of infected cats is always lower than that recorded in dogs living in an endemic area, recent epidemiological studies have suggested that the occurrence of feline leishmaniosis (FeL) might be higher than that currently thought [6]. Despite the increased interest on FeL, little information is available on clinical features, management and treatment of infected cats.

Cats are naturally infected by the same *Leishmania* species affecting dogs and humans worldwide, but progression to active disease is rare and information on adaptive immune response and mechanisms responsible for susceptibility or resistance of feline patients is lacking [7].

Of the few clinical cases reported in the literature, about the half are associated with concurrent immunosuppressive conditions, e.g. feline leukaemia virus (FeLV), feline immunodeficiency virus (FIV), diabetes or neoplasia, thus suggesting that these conditions may act as promoting factors [7]. The most recurrent clinical features in *Leishmania* infected cats are cutaneous lesions including ulcerative, crusty, nodular or scaly dermatitis [5, 7, 8]. These lesions are mainly found on the head and neck and less often on the trunk and legs. The histopathological findings of skin lesions display a diffuse granulomatous dermatitis with macrophages containing many amastigotes forms, or a granulomatous perifolliculitis and lichenoid tissue reaction/interface dermatitis, with a lower parasite load [9]. The most frequent non-cutaneous clinical signs, which have been found alone or in combination, are lymph node enlargement, ocular lesions, gingivostomatitis and decreased appetite [5]. Clinicopathological changes include hyperproteinemia with hypergammaglobulinemia and hypoalbuminemia associated with a reduced albumin/globulin ratio and biochemical abnormalities (e.g. increase of azotemia and hepatic enzymes) [10–12].

Cats affected by FeL are treated with drugs and protocols/dosages prescribed to dogs with the long-term oral administration of allopurinol being the most frequently used treatment [5]. This drug provides clinical improvement and it is generally well tolerated [5]. However, data on clinical signs, pathological alterations, diagnosis, treatment and long-term follow-up are lacking on cats with FeL [13–16].

This study reports clinical, diagnostic and therapeutical findings observed in a domestic shorthair cat with leishmaniosis along with long-term follow-up data, thus providing more evidence-based information on this scantly documented disease of cats.

## Methods

Complete cells blood count, including red blood cells (RBC), haemoglobin (HGB), haematocrit (HCT), white blood cells (WBC) and platelets (PLT), was performed on a K_3_EDTA blood sample using an automated haematology analyser (HeCo Vet C, SEAC, Florence, Italy). Values of serum proteins (i.e. albumin, globulins), creatinine and alanine amino-transferase (ALT) were assessed using commercially available kits by means of an automated UV spectrophotometer (Slim, SEAC). Serum protein fractions were assessed using an automated system (Sel Vet 24, SELEO Engineering, Naples, Italy) according to the manufacturer’s instructions. Infection by FeLV and/or FIV was first tested using an ELISA rapid assay (SNAP Combo FeLV antigen/FIV antibody, IDEXX Laboratories, Westbrook, ME, USA) and further assessed by PCR (FeLV) and nested PCR (FIV) [6]. Smears of the material collected by fine-needle-aspiration of cutaneous lesions were stained using May-Grünwald-Giemsa quick stain (Bio-Optica, Milan, Italy) and microscopically observed at low (200×) and high magnification (1000×). An immunofluorescence antibody test (IFAT) for antibodies against *L. infantum* and real-time PCR (RT-PCR) for parasite kinetoplast DNA from blood and conjunctival swabs were performed as described elsewhere [17, 18].

The parasite was isolated in EMTM and Sloppy Evans medium cultures, and the strain was identified using PCR-restriction fragment length polymorphism and sequencing analysis of ITS1 spacer and the HSP70 gene [19].

## Results

**Fig. 1:**
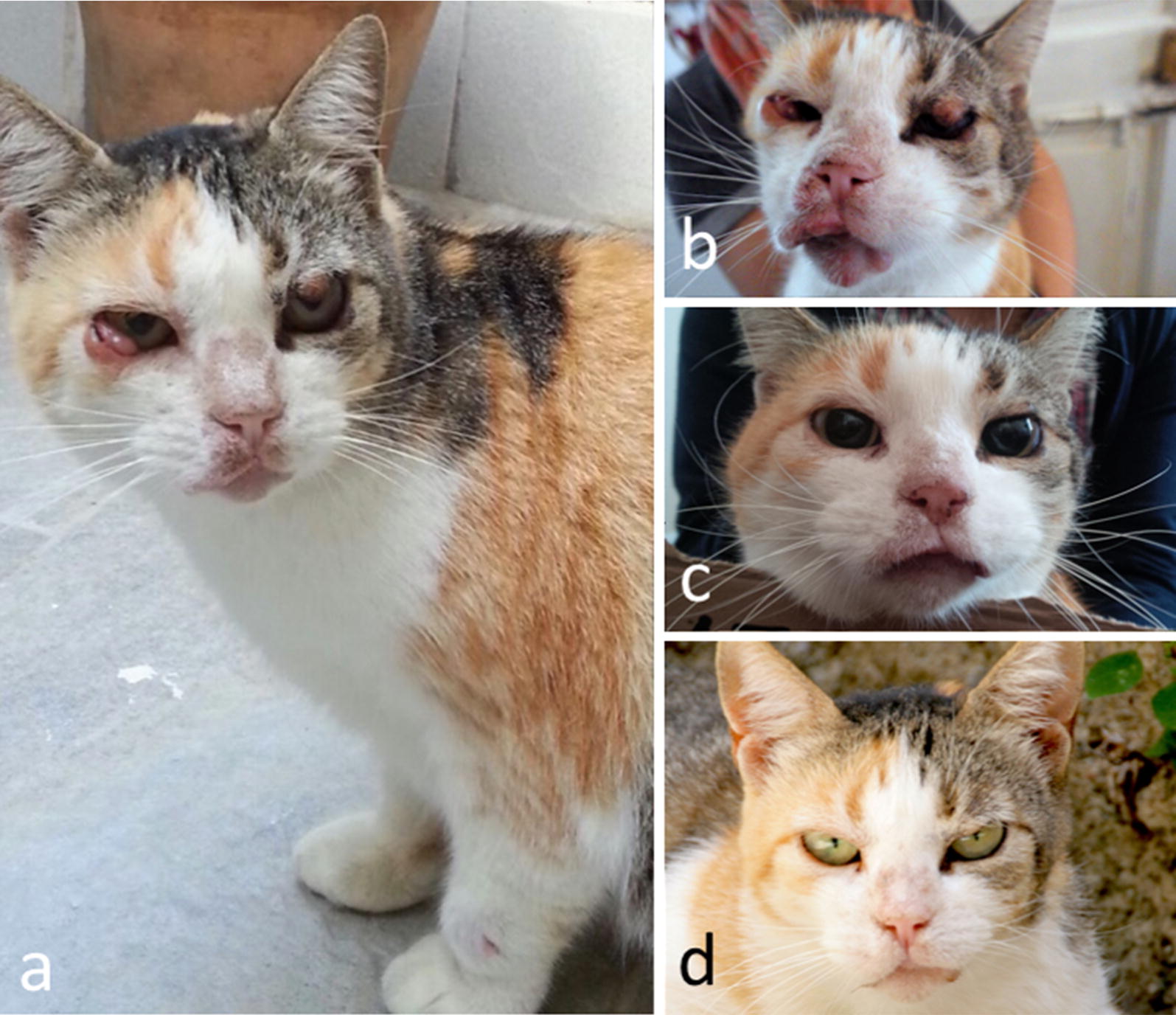
**a** Clinical signs observed in the leishmaniotic cat at the first veterinary examination (October 2014). Note the nodular dermatitis of eyelids on the left carpal region and the nodular conjunctivitis in the right eye. **b** Clinical signs on the face of the cat at the beginning of allopurinol treatment (October 2014). Note the vast ulcerous area of the mucocutaneous junction of the lips. **c** Three-month follow-up. **d** Seven-month follow-up

**Fig. 2:**
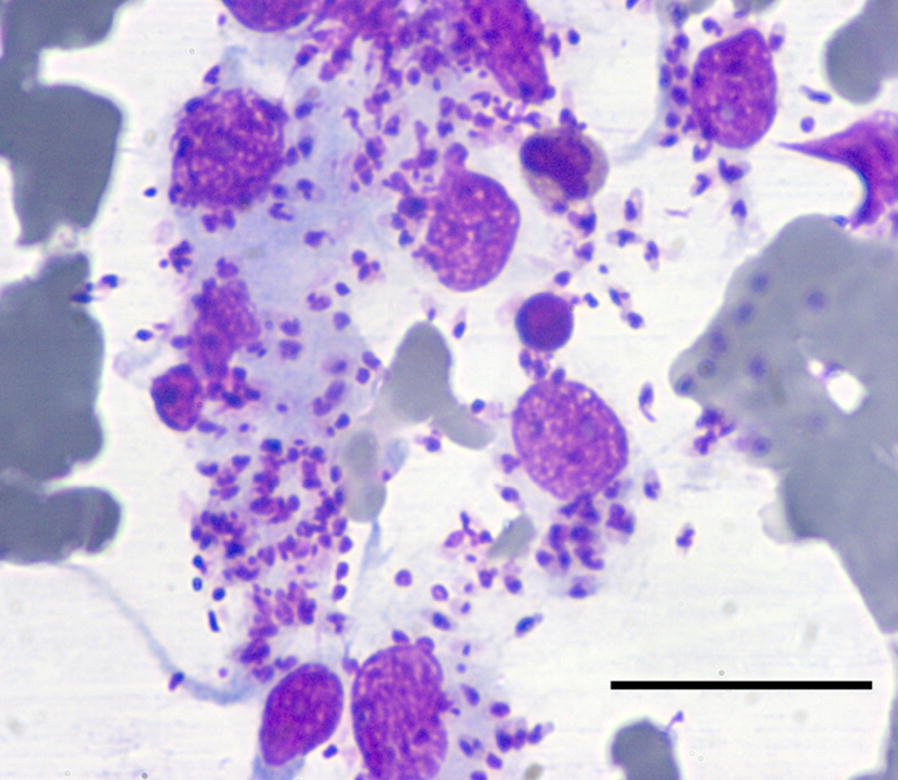
Cytology of the fine needle aspirate of the nodular skin lesion on the eyelid. Note the high load of *Leishmania infantum* amastigotes. May-Grünwald-Giemsa quick stain, 400×. *Scale-bar*: 30 µm

In October 2014, a 6-year-old domestic shorthair female cat living in the urban area of the city of Messina, southern Italy (38°11′39″48N, 15°33′1″80E) was referred to a private veterinary clinic with dermal, oral and ocular alterations. Physical examination revealed areas of nodular dermatitis on the eyelids and left carpal region, blepharitis, conjunctivitis, mucocutaneous ulcerative lesions on the mouth and lymph node enlargement (Fig. 1a, b). The cat was moderately anaemic and hyperglobulinaemic at the complete blood count and serum protein electrophoresis (Table 1), respectively, and tested negative for FeLV and FIV. The initial differential diagnosis included eosinophilic granuloma complex, feline gingivostomatitis complex and neoplasia (e.g. squamous carcinoma or lymphoma) and *Poxvirus* or *Mycobacterium* infections. The cat therefore underwent palliative treatment consisting of antibiotic and corticosteroid (enrofloxacin 5 mg/kg PO SID and prednisone 2.5 mg/kg SID). All the above conditions were excluded according to cytology results, while the presence of numerous macrophages containing intracytoplasmic forms consistent with amastigotes of *Leishmania* (Fig. 2) was documented on fine-needle-aspirates of dermal and mucocutaneous lesions. The cat scored positive to IFAT with a 1:640 IgG titre and positive to RT-PCR on both blood and conjunctival swabs. After the diagnosis of leishmaniosis, palliative treatments were suspended and the cat was treated with days and allopurinol (20 mg/kg PO SID). Three months after commencing allopurinol therapy, nodular dermatitis and conjunctivitis resolved, while ulcerative lesions on the mouth improved significantly (Fig. 1c). Despite the clinical improvement, the cat was persistently positive to *L. infantum* at both serology (1:320) and RT-PCR on blood and conjunctival swabs. Therefore, the treatment was continued with allopurinol at the same dose regime. After seven months, allopurinol was suspended because of a lack of compliance by the owner and due to potential side effect (i.e. intense itch and scratch on the trunk) observed in the last weeks of treatment. Indeed, while potential causes of the itch were excluded (e.g. flea infestation) this sign ceased a few days after the suspension of allopurinol without any other therapy. On this follow-up the cat was apparently healthy (Fig. 1d) but further serological and RT-PCR studies were declined by the owner.

**Table 1:** Haematological and biochemical parameters determined in the leishmaniotic cat at the first veterinary examination before treatment (October 2014), and at the relapse (September 2017)

	October (2014)	September (2017)	Reference range
Haematology^a^			
WBC (×10^3^/µl)	23.4	13.7	6.0–17.0
RBC (×10^6^/µl)	6.2	4.4	5.50–8.5
HCT (%)	27.5	27.1	37–55
HGB (g/dl)	8.9	7.8	12–18
MCV (fl)	44.1	61	60–77
MCH (g/dl)	14.3	17.5	20–25
MCHC (%)	32.4	28.6	32–36
RDW (%)	26.8	16.6	15–27
PLT (×10^3^/µl)	288	183	200–500
Blood chemistry^b^			
Total protein (g/dl)	8.1	9.0	5.4–7.8
Albumin (g/dl)	2.9	2.1	2.1–3.3
Total globulins (g/dl)	5.2	7.9	2.8–5.1
A/G	0.6	0.2	0.6–1.2
Creatinine (mg/dl)	1.1	0.9	0.8–1.8
ALT (U/l)	24	23	6–83

^a^Reference range [18]

^b^Reference range [19]

**Fig. 3:**
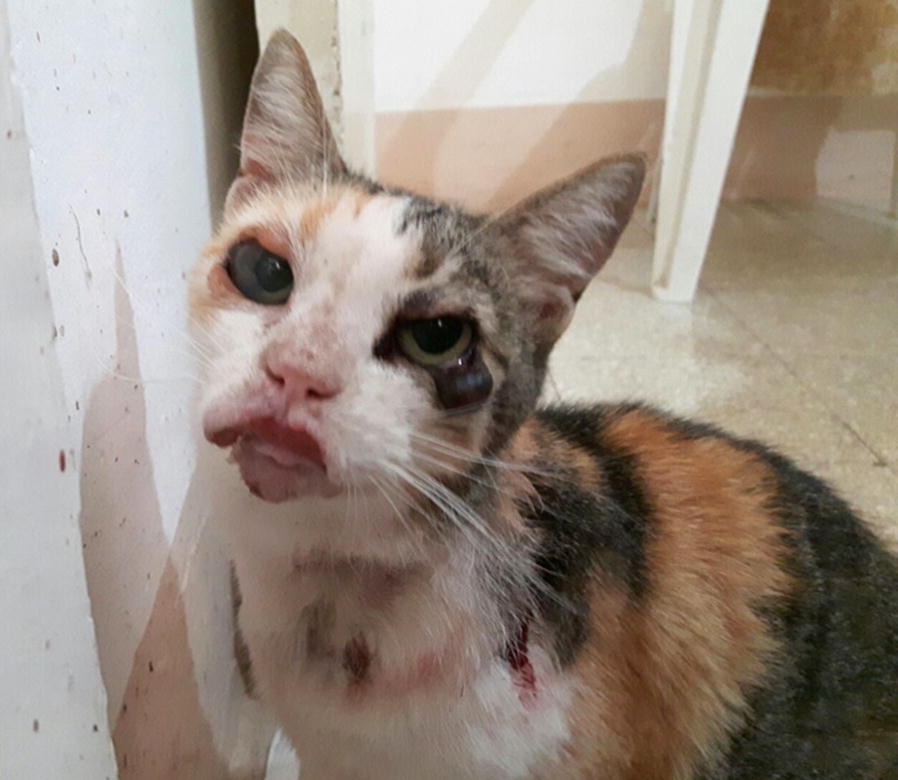
Clinical signs observed in the leishmaniotic cat at the relapse in September 2017

**Fig. 4:**
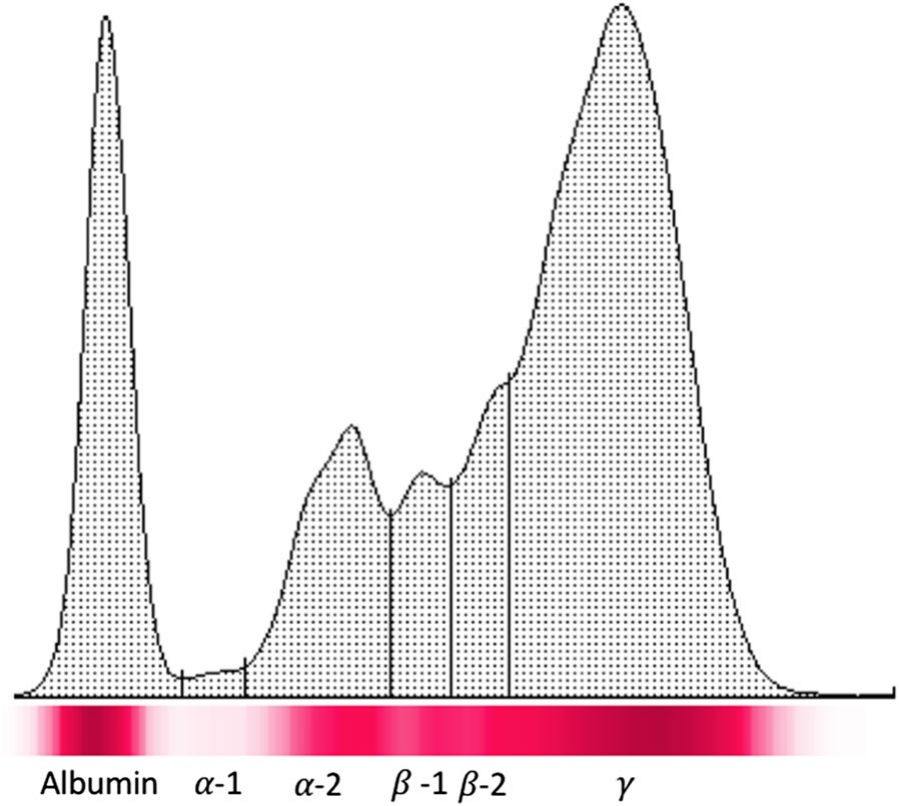
Cellulose acetate electrophoretograms of serum proteins of the leishmaniotic cat at the relapse in September 2017

After two years, in September 2017, the cat relapsed showing almost the same clinical signs upon clinical examination (Fig. 3) and hematological and biochemical abnormalities were observed as well (Table 1). In particular, the cat showed hypochromic and microcytic anaemia, while the protein profile analysis highlighted hyperproteinemia and alteration of electrophoresis (Fig. 4) with hypergammaglobulinemia, hypoalbuminemia and, consequently, a reduced albumin/globulin ratio (0.2) [20, 21].

On this occasion, the parasite was isolated in culture starting from the material collected by fine-needle-aspirate of the nodular lesion on the eyelid, and identified as belonging to *L. infantum*. Unfortunately, the cat owner allowed only domiciliary consultations, and was not compliant in collecting samples (e.g. urine) nor in allowing medical interventions such as sedation or cystocentesis. This impaired the accurate evaluation of alterations and the proper staging of the disease. In addition, despite the suspected adverse reaction observed during the first course of treatment, allopurinol was prescribed again at the same dose regime due to the reluctance of the owner in using other drugs with a complicated route of administration (e.g. subcutaneous injection) or those which were expensive. The treatment with allopurinol was, however, interrupted several times because of the occurrence of the intense itching observed soon after starting this therapy. The cat worsened progressively due to the irregular treatment administration and died two months after the relapse without any chance to shift the treatment to another molecule (e.g. meglumine antimoniate or miltefosine).

## Discussion

Here, we report the clinical signs, pathological findings, allopurinol treatment and 38-month follow-up period of a cat affected by FeL with no other concomitant infections or diseases. Since FeL infection is not usually regarded by practitioners, even in *Leishmania*-endemic areas, its diagnosis is usually not included in the panel of the diagnostic agents for this animal species. The present report, however, confirms the susceptibility of cats to *L. infantum* infection and the progression to disease even in the absence of concurrent immunosuppressive conditions [8]. Significant associations have been found between retroviral infection (i.e. FIV) and FeL, and it has been estimated that about half of the FeL cases reported in literature were associated with impaired immune-competence caused by co-infections or comorbidities [7]. In the present case, FIV and FeLV infections were excluded and cytological examination performed on mucocutaneous ulcerous and nodular dermatitis documented only the presence of numerous *Leishmania* amastigotes and granulomatous reaction. Although the cat was not tested for other vector-borne diseases (i.e. ehrlichiosis, anaplasmosis, bartonellosis), clinical presentation, laboratory abnormalities and an especially good response to specific therapy for leishmaniosis made these co-infections unlikely.

Nodular dermatitis, mucocutanous lesions and ocular disorders are the most frequent signs of clinical FeL usually associated with clinicopathological alterations such as anaemia, leucocytosis, hyperglobulinemia and hypoalbuminemia, as reported for canine leishmaniosis [10–12]. All the above signs and alterations, alone or in combinations, should always alert clinicians to include leishmaniosis in the differential diagnosis process of diseased cats that reside in or have travelled to *Leishmania* endemic areas. The long-term administration of allopurinol was clinically effective resulting in the apparent resolution of the lesions in about seven months. However, the treatment was not effective in curing the infection/eliminating the parasite as demonstrated by molecular and cytological tests in the subsequent follow-ups. It is, however, difficult to assess whether the relapse observed after two years was induced by a reactivation of the previous infection (as suggested by the reappearance of same lesions) or by further re-infections. Indeed, during these two years the cat was not protected with any preventative measure against sand flies and therefore it cannot be excluded that it was subjected to further infective bites. Notably, a matrix collar impregnated with imidacloprid and flumethrin, licensed for the use in cats, has recently proved to be effective in reducing *L. infantum* infection in a cohort of naturally exposed cats [22]. As in dogs, preventative measures by means of repellent products should be adopted for the prevention against sand fly bites and for reducing the risk of *L. infantum* infection [23]. As demonstrated through xenodiagnosis [24], cats with leishmaniosis are infective to sand flies and thus may participate in sustaining the parasite cycle and spreading the disease. Despite the fact that cats are not regarded as a primary reservoir host, the parasitic load in this animal species may be high [25] as observed in this case where a high number of amastigotes was observed in microscopic fields from skin lesions aspirates and successfully used to isolate the parasite in culture. Although the isolation and characterization of *Leishmania* parasites from infected cats are rarely reported [5, 26, 27], the strain herein identified was *L. infantum*, the most common species circulating among dogs, humans and other animal species in the Mediterranean area [2].

Long-term administration of allopurinol is regarded as the most effective treatment for FeL [7]. Although the molecule is generally well tolerated, information on pharmacokinetic and pharmacodynamics as well as safety are lacking for cats. In the present study, itching and scratching were observed and considered as related side effects in the first and especially in the second course of treatment; these side effects were observed soon after starting the therapy. So far, the sole side effects of allopurinol treatment are the elevation of hepatic enzymes and toxicity to kidneys in cats [12, 28], although very recently dermatological signs compatible with a cutaneous adverse drug reaction were described in a *L. infantum*-positive cat treated with allopurinol [29]. Therefore, even though allopurinol currently has the most extensive clinical experience available for FeL, its administration to cats needs to be strictly monitored and fine-tuned according to clinical response, owner compliance and safety.

The case herein reported describes the course of a FeL case that survived 38 months after the first diagnosis with a well-maintained quality of life, except in the last months after the relapse in which it worsened rapidly despite treatment attempts. According to a recent retrospective evaluation of 14 cases of FeL, the median survival time is three months after the first diagnosis and it seems that there are no significant differences among treated cats (median time five months), not treated (median time one month) and FIV co-infected (median two and a half months). Therefore, the expectancy of life of FeL patients is not significantly influenced by therapy or retroviral coinfection [12]. However, treatment of FeL should be always attempted since it may provide a better quality of life to diseased animals, a longer survival time and a significant reduction of the parasite load which, in turn, may result in a lower infectivity to sand flies.

## Conclusions

This study further confirms the potential progression of *Leishmania* infection to disease in a cat patient even in the absence of comorbidities. Although the long-term allopurinol treatment provided an improvement of the clinical manifestation, it was unsuccessful in controlling the disease. In addition, despite the drug being well tolerated, side effects may appear and the patients should be systematically monitored during the treatment course. Veterinarians practicing in endemic areas should be aware of the susceptibility of cats to *Leishmania* infection, properly include FeL in the differential diagnosis and propose preventative measures to those cats at risk.

## Abbreviations

FeL: feline leishmaniosis
FeLV: feline leukemia virus
FIV: feline immunodeficiency virus
PCR: polymerase chain reaction
IFAT: immunofluorescence antibody test
EMTM: Evans’ modified Tobie’s medium
